# A bacterial PriB with weak single-stranded DNA binding activity can stimulate the DNA unwinding activity of its cognate PriA helicase

**DOI:** 10.1186/1471-2180-11-189

**Published:** 2011-08-23

**Authors:** Cui Feng, Bharath Sunchu, Mallory E Greenwood, Matthew E Lopper

**Affiliations:** 1 Department of Chemistry, University of Dayton, 300 College Park, Dayton, OH 45469, USA; 2 College of Pharmacy, University of Illinois at Chicago, 833 S. Wood St., Chicago, Il 60612, USA

## Abstract

**Background:**

Bacterial DNA replication restart pathways facilitate reinitiation of DNA replication following disruptive encounters of a replisome with DNA damage, thereby allowing complete and faithful duplication of the genome. In *Neisseria gonorrhoeae*, the primosome proteins that catalyze DNA replication restart differ from the well-studied primosome proteins of *E. coli *with respect to the number of proteins involved and the affinities of their physical interactions: the PriA:PriB interaction is weak in *E. coli*, but strong in *N. gonorrhoeae*, and the PriB:DNA interaction is strong in *E. coli*, but weak in *N. gonorrhoeae*. In this study, we investigated the functional consequences of this affinity reversal.

**Results:**

We report that *N. gonorrhoeae *PriA's DNA binding and unwinding activities are similar to those of *E. coli *PriA, and *N. gonorrhoeae *PriA's helicase activity is stimulated by its cognate PriB, as it is in *E. coli*. This finding is significant because *N. gonorrhoeae *PriB's single-stranded DNA binding activity is weak relative to that of *E. coli *PriB, and in *E. coli*, PriB's single-stranded DNA binding activity is important for PriB stimulation of PriA helicase. Furthermore, a *N. gonorrhoeae *PriB variant defective for binding single-stranded DNA can stimulate PriA's helicase activity, suggesting that DNA binding by PriB might not be important for PriB stimulation of PriA helicase in *N. gonorrhoeae*. We also demonstrate that *N. gonorrhoeae *PriB stimulates ATP hydrolysis catalyzed by its cognate PriA. This activity of PriB has not been observed in *E. coli*, and could be important for PriB stimulation of PriA helicase in *N. gonorrhoeae*.

**Conclusions:**

The results of this study demonstrate that a bacterial PriB homolog with weak single-stranded DNA binding activity can stimulate the DNA unwinding activity of its cognate PriA helicase. While it remains unclear if *N. gonorrhoeae *PriB's weak DNA binding activity is required for PriB stimulation of PriA helicase, the ability of PriB to stimulate PriA-catalyzed ATP hydrolysis could play an important role. Thus, the weak interaction between *N. gonorrhoeae *PriB and DNA might be compensated for by the strong interaction between PriB and PriA, which could result in allosteric activation of PriA's ATPase activity.

## Background

DNA damage contributes to genome instability by creating barriers that hinder the progression of the replication machinery (replisome) during DNA replication [1]. Consequently, DNA replication forks that stall or collapse due to encounters of the replisome with DNA damage must be reactivated to allow complete replication of the genome and ensure survival of the cell. DNA replication restart pathways provide bacterial cells with a mechanism to reactivate replisomes that are disrupted in this manner [2]. Catalyzed by primosome proteins such as PriA, PriB, PriC, DnaT, and DnaG, DNA replication restart pathways facilitate origin-independent reloading of the replicative helicase onto a repaired DNA replication fork in a process that involves coordinated protein and nucleic acid binding within a nucleoprotein complex called the DNA replication restart primosome [2].

DNA replication restart initiated by PriA helicase is a highly coordinated process and probably represents the major pathway of DNA replication restart in *E. coli*. PriA belongs to the DExH family of DNA helicases and is well-conserved among sequenced bacterial genomes [3]. PriA is thought to recognize and bind to repaired DNA replication forks and D-loop recombination intermediates, facilitate assembly of the primosome complex by recruiting other primosome proteins, and catalyze duplex DNA unwinding using energy furnished by hydrolysis of ATP [4,5]. Recruitment of PriB to a PriA:DNA complex stabilizes PriA on the DNA [6] and enhances its helicase activity through a mechanism that involves PriB's single-stranded DNA-binding activity [7]. Formation of a PriA:PriB:DNA complex leads to recruitment of DnaT, perhaps through physical interactions with PriB [6]. The function of DnaT is not well understood, but it has been proposed that DnaT binding leads to dissociation of single-stranded DNA (ssDNA) from PriB through a competition mechanism, possibly exposing the ssDNA on the lagging strand template for reloading the replicative helicase, which ultimately leads to fork reactivation [8].

While studies of DNA replication restart pathways have focused primarily on the well-studied *E. coli *model organism, DNA replication restart has been shown to be important in other bacteria as well, including the medically important bacterium, *Neisseria gonorrhoeae. N. gonorrhoeae *is a gram-negative bacterium and the causative agent of gonorrhea. Infections are associated with a host inflammatory response that is mounted against the pathogen involving phagocytic cells such as polymorphonuclear granulocytes [9]. The ability of phagocytes to produce reactive oxygen species as an antimicrobial mechanism has been well-established, and commensal organisms such as lactobacillus species have been shown to produce and secrete H_2_O_2_, thus making it likely that *N. gonorrhoeae *faces considerable oxidative challenges in infected individuals [10,11].

A variety of studies have examined the sensitivity of *N. gonorrhoeae *to oxidative stress. Among them, one has demonstrated that *N. gonorrhoeae *can utilize enzymatic mechanisms such as catalase, peroxidase, and glutathione to protect against reactive oxygen species [12], another has shown that manganese is important for chemically scavenging superoxide [13], and yet another has revealed a role for DNA recombination and repair enzymes such as RecA, RecBCD, and enzymes of the RecF-like pathway in resistance to oxidative stress [14]. In addition, PriA has been shown to play a critical role in DNA repair and in resisting the toxic effects of oxidative damaging agents, suggesting that DNA replication restart pathways might play an important role in *N. gonorrhoeae *resistance to oxidative stress and overall pathogenicity [15]. This notion is further supported by observations that *priA *from a related bacterium, *Neisseria meningitidis*, is an important virulence determinant and is important for resisting oxidative injury and promoting bacterial replication [16].

Studies of DNA replication restart pathways in diverse bacteria such as *E. coli *and *N. gonorrhoeae *have revealed species differences in the composition of the DNA replication restart primosome and in the functions of the individual primosome proteins. For example, *N. gonorrhoeae *lacks a recognizable homolog of *dnaT *in its genome, suggesting that the *N. gonorrhoeae *PriA-PriB pathway might be significantly different from the *E. coli *PriA-PriB-DnaT pathway. Furthermore, physical interactions between primosome components show variation in their individual binary affinities: the physical interaction between PriA and PriB is rather weak in *E. coli*, but relatively strong in *N. gonorrhoeae*, and the physical interaction between PriB and ssDNA is strong in *E. coli*, but relatively weak in *N. gonorrhoeae *[8,17,18]. Thus, the affinities of binary interactions between primosome components are reversed between the two species.

Since the ssDNA-binding activity of PriB is important for PriB-stimulation of PriA's helicase activity in *E. coli *[7], there might be significant functional consequences for the variation in affinities of physical interactions within the *N. gonorrhoeae *PriA-PriB primosome. In this study, we investigated the functional consequences of the affinity reversal phenomenon by examining the helicase activity of *N. gonorrhoeae *PriA, and we determined how PriA-catalyzed ATP hydrolysis and DNA unwinding are affected by *N. gonorrhoeae *PriB.

## Results

### DNA binding by PriA, but not PriB, is structure-specific

We used fluorescence polarization spectroscopy to examine the physical interaction between *N. gonorrhoeae *PriA and a variety of DNA structures that were constructed by annealing fluorescein-labeled synthetic DNA oligonucleotides. The DNA structures include ssDNA, a partial duplex DNA with a 3' ssDNA overhang, and a forked DNA structure with fully duplex leading and lagging strand arms (Table 1). The presence of a fluorescein tag on the DNAs allowed us to measure PriA binding to the DNA due to the increase in fluorescence polarization of the PriA:DNA complex relative to the unbound DNA. PriA protein was serially diluted and incubated with 1 nM fluorescein-labeled DNA and the fluorescence polarization was measured. Apparent dissociation constants were obtained by determining the concentration of PriA needed to achieve 50% binding to each of the various DNA substrates.

**Table 1:** DNA substrates.

ssDNA		**oML228: **5'-AAG CAC AAT TAC CCA CGC
dsDNA		**oML230: **5'-GCC GTG ATC ACC AAT GCA GAT TGA CGA ACC TTT GCC **oML233: **5'-GGC AAA GGT TCG TCA ATC TGC ATT GGT GAT CAC GGC

3' Overhang		**oML276: **5'-AAC GTC ATA GAC GAT TAC ATT GCT ACA TGG AGC TGT CTA GAG GAT CCG AC **oML277: **5'-TAG CAA TGT AAT CGT CTA TGA CGT T

Fork 1		**oML211: **5'-GTC GGA TCC TCT AGA CAG CTC CAT GAT CAC TGG CAC TGG TAG AAT TCG GC **oML212: **5'-GCC GAA TTC TAC CAG TGC CAG TGA T **oML287: **5'-ACG ATT ACA TTG CTA CAT GGA GCT GTC TAG AGG ATC CGA C **oML288: **5'-TAG CAA TGT AAT CGT

Fork 2		

Fork 3		**oML213: **5'-ACG TAG GCC GGA AAC AAC GTC ATA GAC GAT TAC ATT GCT ACA TGG AGC TGT CTA GAG GAT CCG AC **oML278: **5'-TAG CAA TGT AAT CGT CTA TGA CGT TGT TTC CGG CCT ACG T

The 3' end of each DNA oligonucleotide is denoted by a half arrow. The asterisk denotes the position of the fluorescein label. Numbers in parentheses denote the number of bases in the oligonucleotide.

**Figure 1:**
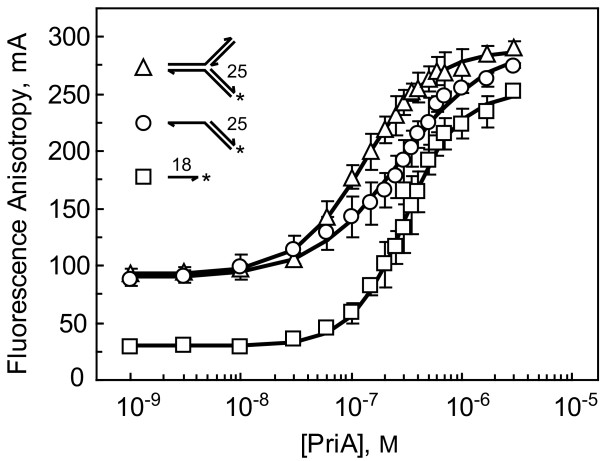
**DNA binding activity of *N. gonorrhoeae *PriA**. PriA was serially diluted and incubated with 1 nM fluorescein-labeled ssDNA (squares), 3' Overhang (circles), or Fork 2 (triangles). Measurements are reported in triplicate and error bars represent one standard deviation of the mean.

As expected based on studies of *E. coli *PriA DNA binding [5,19-22], *N. gonorrhoeae *PriA binds each of the DNA structures that we tested (Figure 1). PriA binds the forked DNA structure (Fork 2) with the highest affinity of the DNA structures tested, resulting in an apparent dissociation constant of 134 ± 22 nM (Table 2). This DNA structure has fully duplex leading and lagging strand arms with no gap at the three-way junction, and a hydroxyl group exists at the 3' end of the leading strand arm to provide contacts with the 3' hydroxyl binding pocket of PriA's DNA binding domain, assuming that this feature of the helicase has been conserved between the *E. coli *and *N. gonorrhoeae *homologs [23].

**Table 2:** Apparent dissociation constants for PriA:DNA and PriB:DNA complexes.

DNA Substrate	PriA K_d,app, _nM	PriB K_d,app, _nM
ssDNA	307 ± 43	662 ± 37
dsDNA	ND	640 ± 35
3' Overhang	234 ± 62	628 ± 95
Fork 2	134 ± 22	690 ± 51

Apparent dissociation constants (K_d,app_) are mean values derived from at least three independent experiments and associated uncertainty values are one standard deviation of the mean. ND: Not determined.

The apparent dissociation constants for the partial duplex DNA with a 3' ssDNA overhang and the ssDNA substrate are higher than that of the forked DNA substrate, with values of 234 ± 62 nM (3' Overhang) and 307 ± 43 nM (ssDNA) (Table 2). While we can not rule out the possibility that the differences in affinity are due to differences in the size of the DNA substrates, it is possible that the partial duplex DNA and the ssDNA substrates lack structural elements that are needed to achieve the high affinity binding observed with the forked DNA substrate.

Work from several laboratories has demonstrated that *E. coli *PriB is a ssDNA-binding protein [18,24-27], and previous work from our laboratory has shown that *N. gonorrhoeae *PriB binds ssDNA, albeit with a significantly lower affinity than does the *E. coli *PriB homolog [17]. Despite this lower affinity, *N. gonorrhoeae *PriB has the structural hallmark of a ssDNA-binding protein [17], leading us to hypothesize that it would bind ssDNA and any DNA structures that contain ssDNA with higher affinity than duplex DNAs. To test this hypothesis, we used fluorescence polarization spectroscopy to measure PriB's DNA binding activity using a variety of DNA structures, including ssDNA, double-stranded DNA (dsDNA), a partial duplex DNA with a 3' ssDNA overhang, and a forked DNA structure with fully duplex leading and lagging strand arms (Table 1).

**Figure 2:**
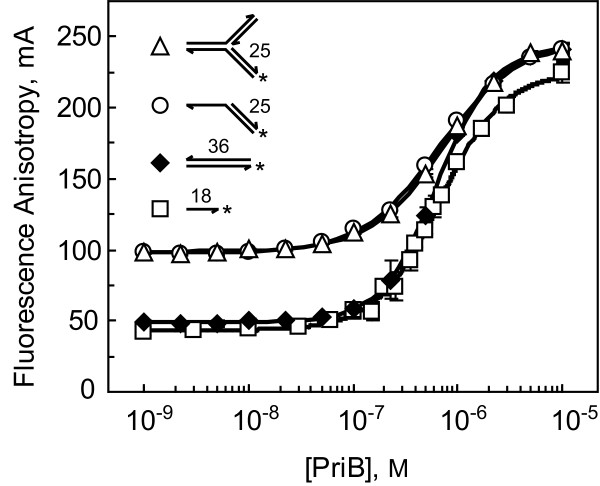
**DNA binding activity of *N. gonorrhoeae *PriB**. PriB was serially diluted and incubated with 1 nM fluorescein-labeled ssDNA (squares), dsDNA (closed diamonds), 3' Overhang (circles), or Fork 2 (triangles). Measurements are reported in triplicate and error bars represent one standard deviation of the mean.

Contrary to expectations, the DNA binding activity of PriB shows little preference for specific DNA structures (Figure 2). The apparent dissociation constants range from 628 ± 95 nM (3' Overhang) to 690 ± 51 nM (Fork 2), and the observed differences among apparent dissociation constants for the various DNA structures are insignificant given the experimental uncertainty of the measurements (Table 2). This observation, together with the low affinity with which *N. gonorrhoeae *PriB binds DNA relative to *E. coli *PriB, suggests that the surface of this PriB homolog might have been adapted for a purpose other than binding ssDNA. Furthermore, it raises the important question of whether *N. gonorrhoeae *PriB can stimulate its cognate PriA's helicase activity, since in *E. coli *this stimulatory effect depends on PriB's strong ssDNA-binding activity [7].

### PriA helicase activity is limited to short stretches of duplex DNA

To test the functional consequences of *N. gonorrhoeae *PriB's weak DNA binding activity, we first had to examine *N. gonorrhoeae *PriA's helicase activity. We used the partial duplex and forked DNA structures shown in Table 1 as substrates based on extensive studies of substrate preference and helicase activity of *E. coli *PriA [22,28,29]. For each of these substrates, the fluorescein-labeled strand represents the nascent lagging strand arm, and the degree of duplex DNA unwinding of the fluorescein-labeled strand was determined using fluorescence polarization spectroscopy. For these experiments, the DNA substrates were incubated with PriA and ATP for 10 min at 37°C, the reactions were terminated by addition of SDS, and the fluorescence polarization of the samples was measured. The degree of unwinding was determined by comparing the fluorescence polarization of the samples to that of the DNA substrate incubated in buffer alone (fully intact DNA substrate) and to the samples heated briefly to 95°C and fast-cooled back to 25°C (fully denatured DNA substrate). This allowed us to measure the fraction of each DNA substrate that is unwound by various concentrations of PriA.

**Figure 3:**
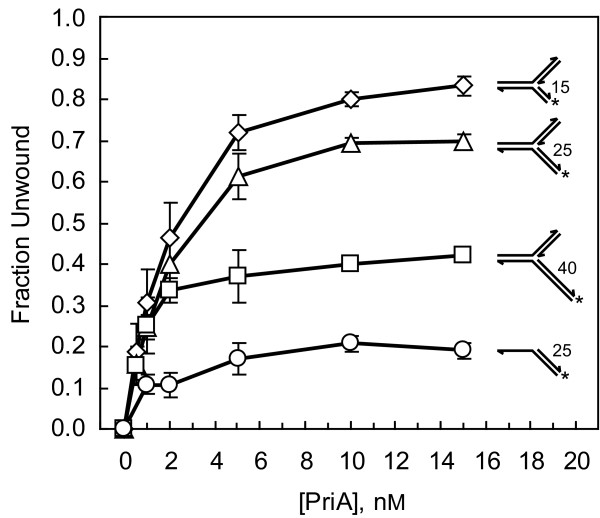
**Helicase activity of *N. gonorrhoeae *PriA**. PriA-catalyzed duplex DNA unwinding was examined using 1 nM Fork 1 (15 bp lagging strand arm, diamonds), Fork 2 (25 bp lagging strand arm, triangles), Fork 3 (40 bp lagging strand arm, squares), or 3' Overhang (25 bp partial duplex, circles). Measurements are reported in triplicate and error bars represent one standard deviation of the mean.

Of the DNA substrates examined, PriA shows greatest unwinding activity on forked DNA substrates with relatively short duplex lagging strand arms. Levels of maximal unwinding are approximately 83% for Fork 1 (15 bp lagging strand arm), 70% for Fork 2 (25 bp lagging strand arm), and 42% for Fork 3 (40 bp lagging strand arm) (Figure 3). PriA shows significantly lower unwinding activity on the partial duplex DNA with a 3' ssDNA overhang. Maximal unwinding activity is approximately 19% for this substrate, suggesting that the partial duplex DNA lacks structural elements required for efficient PriA binding and unwinding (Figure 3). This has been observed for *E. coli *PriA helicase as well [7,28]. Overall, these results demonstrate that *N. gonorrhoeae *PriA helicase activity is limited to relatively short stretches of duplex DNA, akin to its *E. coli *counterpart.

Comparison of the helicase activity of *N. gonorrhoeae *PriA that we measured in this study with the previously reported helicase activity of *E. coli *PriA at the same concentrations and on similar DNA substrates reveals that the two PriA homologs follow the same trend with respect to the dependence of their DNA unwinding activity on the length of the duplex arm of the DNA substrate (Table 3). There are some differences in the degree of DNA unwinding catalyzed by *N. gonorrhoeae *PriA that we measured in this study compared with the helicase activity previously reported for *E. coli *PriA. For example, *E. coli *PriA helicase shows slightly elevated DNA unwinding activity on the 25 bp fork structure compared to *N. gonorrhoeae *PriA (Table 3). Whether this represents natural biological variation between the two PriA homologs or differences arising from work involving separate investigators is uncertain.

**Table 3:** Comparison of helicase activity of *E. coli *PriA and *N. gonorrhoeae *PriA.

DNA Substrate	*E. coli *PriA^1 ^% DNA Unwound	*N. gonorrhoeae *PriA^2 ^% DNA Unwound
25 bp fork	83 ± 3	61 ± 6
40 bp fork	28 ± 8	37 ± 7
25 bp partial duplex	23 ± 2	17 ± 4

^1^Cadman *et al*. J Biol Chem 2005, **280**(48):39693-39700.

^2^This study.

In this study, the 25 bp fork substrate is Fork 2, the 40 bp fork substrate is Fork 3, and the 25 bp partial duplex substrate is 3' Overhang. The helicase activity for each PriA homolog is the mean percent of DNA unwound by 5 nM PriA on 1 nM DNA substrate and in the absence of its cognate PriB. Mean values from Cadman *et al*. are derived from two independent experiments, and mean values from this study are derived from three independent experiments. Associated uncertainty values are one standard deviation of the mean.

### PriB stimulates PriA's helicase activity on long regions of duplex DNA

**Figure 4:**
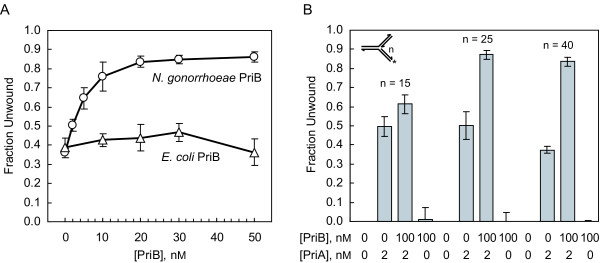
**PriB stimulates the helicase activity of PriA**. A) Unwinding of 1 nM Fork 3 by 2 nM PriA in the presence of *N. gonorrhoeae *PriB (circles) or *E. coli *PriB (triangles). Measurements are reported in triplicate and error bars represent one standard deviation of the mean. B) Unwinding of 1 nM forked DNA substrates by 2 nM PriA in the presence or absence of 100 nM *N. gonorrhoeae *PriB (as monomers). The inset shows the structure of the DNA substrates, where *n *equals the length of the fluorescein-labeled lagging strand arm. Measurements are reported in triplicate and error bars represent one standard deviation of the mean.

To determine if *N. gonorrhoeae *PriB stimulates the helicase activity of its cognate PriA, we examined PriA helicase activity on a forked DNA substrate with a 40 bp lagging strand arm (Fork 3) in the presence and absence of PriB. Under these experimental conditions, incubating 2 nM PriA with Fork 3 in the absence of PriB results in 36% unwinding (Figure 4A). In the presence of PriB, the maximal degree of unwinding is approximately 86%, with near saturating unwinding activity obtained with 20 nM PriB (as monomers). This represents an approximately 2.4 fold stimulation of PriA helicase activity by PriB. Increasing the concentration of PriB to 100 nM (as monomers) does not significantly increase the fold stimulation of PriA helicase activity on this DNA substrate (Figure 4B). *E. coli *PriB fails to stimulate *N. gonorrhoeae *PriA helicase activity on Fork 3, indicating that PriB stimulation of PriA helicase activity is species-specific (Figure 4A), and duplex DNA unwinding by PriB is negligible in the absence of PriA, indicating that PriB stimulation of PriA helicase activity is not due to a helicase contaminant in the PriB preparation (Figure 4B).

We also examined PriB's ability to stimulate PriA helicase activity on forked DNA substrates with relatively shorter lagging strand arms. Using 2 nM PriA, we observed a 1.2 fold stimulation of PriA helicase activity on a forked DNA substrate with a 15 bp lagging strand arm (Fork 1), and a 1.7 fold stimulation of PriA helicase activity on a forked DNA substrate with a 25 bp lagging strand arm (Fork 2) (Figure 4B). Therefore, while the overall degree of PriA-catalyzed duplex DNA unwinding decreases as the length of the lagging strand arm increases, the relative stimulatory effect of PriB increases (Tables 3 and 4). This same trend is observed for PriB stimulation of PriA helicase activity in *E. coli *[7].

**Table 4:** Comparison of PriB stimulation of PriA helicase activity in *E. coli *and *N. gonorrhoeae*.

DNA Substrate	*E. coli*^1 ^Fold Stimulation of PriA by PriB	*N. gonorrhoeae*^2 ^Fold Stimulation of PriA by PriB
15 bp fork	ND	1.2
25 bp fork	1.0	1.7
40 bp fork	2.6	2.4
50 bp fork	10.4	ND
60 bp fork	10.8	ND
70 bp fork	~ 9	ND

^1^Cadman *et al*. J Biol Chem 2005, 280(48):39693-39700.

^2^This study.

In this study, the 15 bp fork substrate is Fork 1, the 25 bp fork substrate is Fork 2, and the 40 bp fork substrate is Fork 3. The fold stimulation of PriA helicase activity by PriB is the ratio of the level of unwinding of the DNA substrate by PriA in the presence versus the absence of PriB. In Cadman *et al*., stimulation of *E. coli *PriA helicase by its cognate PriB was assayed using 200 nM PriB monomers and 5 nM PriA (20-fold excess of PriB dimers to PriA). In this study, stimulation of *N. gonorrhoeae *PriA helicase by its cognate PriB was assayed using 100 nM PriB monomers and 2 nM PriA (25-fold excess of PriB dimers to PriA). ND: Not determined.

We compared the fold stimulation of *N. gonorrhoeae *PriA helicase activity by PriB that we measured in this study with that previously reported for *E. coli *PriA and PriB and found that the fold stimulation is similar for a 40 bp duplex fork structure. In *E. coli*, PriB stimulates PriA helicase activity 2.6 fold on the 40 bp duplex fork structure, and *N. gonorrhoeae *PriB stimulates PriA helicase activity 2.4 fold on the same DNA substrate (Table 4). There is a slight difference between the *E. coli *and *N. gonorrhoeae *proteins on a 25 bp duplex fork structure. On this DNA substrate, *N. gonorrhoeae *PriB stimulates PriA helicase activity 1.7 fold, while *E. coli *PriB does not stimulate PriA helicase activity to a significant degree (Table 4). While the significance of this is unclear, it could be attributed to the relatively lower levels of DNA unwinding by *N. gonorrhoeae *PriA on this DNA substrate in the absence of PriB compared to that catalyzed by *E. coli *PriA, thus permitting a greater degree of stimulation of *N. gonorrhoeae *PriA helicase activity when PriB is present.

**Figure 5:**
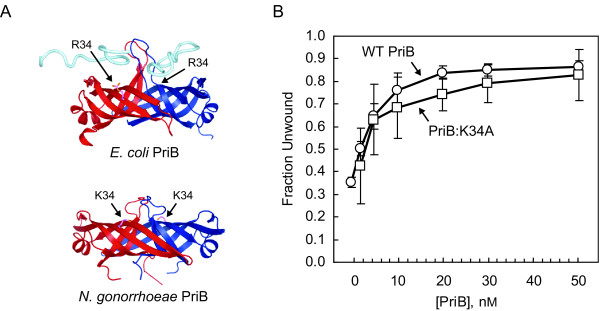
**A PriB variant defective for ssDNA binding stimulates the helicase activity of PriA**. A) Ribbon diagrams of the crystal structures of *E. coli *PriB complexed with ssDNA (top, PDB code 2CCZ) and *N. gonorrhoeae *PriB (bottom, PDB code 3K8A). The two monomers of the PriB dimers are colored red and blue, and the ssDNA is rendered as a cyan tube. The ssDNA modeled above the red chain of *E. coli *PriB is derived from a symmetry-related molecule in the crystal structure. Amino acid residue K34 of *N. gonorrhoeae *PriB, and the structurally-analogous R34 amino acid residue of *E. coli *PriB are rendered as sticks with carbon atoms colored violet and nitrogen atoms colored orange. B) Unwinding of 1 nM Fork 3 by 2 nM PriA in the presence of wild type *N. gonorrhoeae *PriB (circles) or PriB:K34A (squares). Measurements are reported in triplicate and error bars represent one standard deviation of the mean.

We were surprised to observe that *N. gonorrhoeae *PriB has a stimulatory effect on the DNA unwinding activity of PriA because in *E. coli*, stimulation of PriA helicase by PriB involves PriB's ssDNA binding activity [7], which is relatively weak in *N. gonorrhoeae *PriB [17]. Therefore, we tested the ability of a *N. gonorrhoeae *PriB variant, PriB:K34A, to stimulate the DNA unwinding activity of its cognate PriA. Amino acid residue K34 of *N. gonorrhoeae *PriB maps to the ssDNA binding site and is structurally analogous to residue R34 of *E. coli *PriB, which is involved in binding ssDNA (Figure 5A) [26]. The PriB:K34A variant is defective for ssDNA binding, and a lower limit for the apparent dissociation constant for the interaction of PriB:K34A with ssDNA has been estimated at > 3 μM [17]. The actual dissociation constant could be much higher, but PriB:K34A fails to reach saturable ssDNA binding at the highest protein concentrations that were used in the equilibrium DNA binding assays that were previously reported for this PriB variant [17].

When we examined PriA helicase activity on Fork 3 in the presence of PriB:K34A, we found that levels of DNA unwinding are similar to those seen when wild type PriB is used to stimulate PriA (Figure 5B). Based on the value of the apparent dissociation constant for the interaction of PriB:K34A with ssDNA, and assuming that it is a reliable indicator of the affinity of PriB:K34A for DNA in the context of a ternary PriA:PriB:DNA complex, we would not expect the PriB:K34A variant to be interacting with DNA to a significant degree under the conditions of this DNA unwinding assay. It is particularly noteworthy that in *E. coli*, a PriB variant with severely weakened ssDNA binding activity (the W47,K82A double mutant) fails to stimulate the DNA unwinding activity of its cognate PriA to a significant degree [7]. Therefore, unless formation of a PriA:PriB:DNA ternary complex significantly enhances the DNA binding activity of *N. gonorrhoeae *PriB, our results suggest that ssDNA binding by *N. gonorrhoeae *PriB does not play a major role in *N. gonorrhoeae *PriB stimulation of its cognate PriA helicase.

### PriB activates PriA's ATPase activity

**Figure 6:**
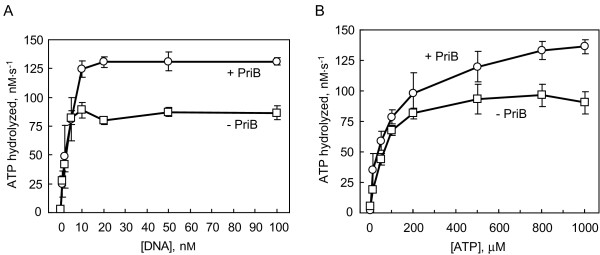
**PriA's ATPase activity is stimulated by DNA and by PriB**. A) DNA-dependent ATP hydrolysis catalyzed by 10 nM PriA in the presence (circles) or absence (squares) of 100 nM PriB (as monomers). The DNA substrate is Fork 3. Measurements are reported in triplicate and error bars represent one standard deviation of the mean. B) Effect of ATP concentration on rates of ATP hydrolysis catalyzed by 10 nM PriA in the presence of 100 nM Fork 3 and in the presence (circles) or absence (squares) of 100 nM PriB (as monomers). Measurements are reported in triplicate and error bars represent one standard deviation of the mean.

PriA helicase is thought to couple the energy released from hydrolysis of ATP to the unwinding of duplex DNA. Thus, we wanted to determine if *N. gonorrhoeae *PriB stimulation of PriA helicase activity involves PriA's ability to hydrolyze ATP. To examine PriA's ATPase activity, we used a spectrophotometric assay that couples PriA-catalyzed ATP hydrolysis to oxidation of NADH. This assay allowed us to measure steady-state PriA-catalyzed ATP hydrolysis rates in the presence and absence of PriB. As expected, PriA's ATPase activity is negligible in the absence of DNA (Figure 6A). The DNA dependence of PriA's ATPase activity has been observed in *E. coli *as well [30], and likely reflects a mechanistic coupling of ATP hydrolysis and duplex DNA unwinding.

With 10 nM PriA and in the absence of PriB, near maximal rates of ATP hydrolysis are observed with 10 nM Fork 3 (Figure 6A). Under these experimental conditions, the *K*_m _with respect to DNA is 2 ± 1 nM and the *k*_cat _is 9 ± 1 s^-1 ^(Table 5). To determine if PriB affects the ATPase activity of PriA, we measured ATP hydrolysis catalyzed by 10 nM PriA in the presence of 100 nM PriB (as monomers) and various concentrations of Fork 3 DNA (Figure 6A). This produces the same ratio of PriB to PriA that results in near maximal stimulation of PriA helicase activity (Figure 4A). Addition of 100 nM PriB (as monomers) yields a *K*_m _with respect to DNA of 3 ± 1 nM (Table 5). Thus, the presence of PriB has no significant effect on PriA's *K*_m _with respect to DNA. We also examined the effect of PriB on PriA's *K*_m _with respect to ATP (Figure 6B). With 10 nM PriA and in the absence of PriB, the *K*_m _with respect to ATP is 54 ± 19 μM (Table 5). Addition of 100 nM PriB (as monomers) yields a *K*_m _with respect to ATP of 70 ± 13 μM (Table 5). Thus, the presence of PriB has no significant effect on PriA's *K*_m _with respect to ATP.

**Table 5:** Kinetic parameters for PriA's ATPase activity in the presence and absence of PriB.

	- PriB	+ PriB
***K*_m,DNA, _nM**	2 ± 1	3 ± 1
***K*_m,ATP_, μM**	54 ± 19	70 ± 13
***k*_cat_, s^-1^**	9 ± 1	14 ± 1

Kinetic parameters are mean values derived from at least three independent experiments and associated uncertainty values are one standard deviation of the mean.

While PriB does not have a significant effect on PriA's *K*_m _values for ATP or DNA, it does have a significant effect on the value of *k*_cat_. In the presence of 100 nM PriB (as monomers), the *k*_cat _increases to 14 ± 1 s^-1^, indicating that PriB activates PriA's ATPase activity (Figure 6 and Table 5). This observation lies in contrast to studies performed using *E. coli *PriA and PriB proteins that reveal no effect of PriB on the rate of PriA-catalyzed ATP hydrolysis [7].

## Discussion

In this study, we examined physical interactions within the DNA replication restart primosome of *N. gonorrhoeae *and the functional consequences of those interactions to gain insight into the biological significance of species variation in primosome protein function. Physical interactions within the DNA replication restart primosome of *N. gonorrhoeae *differ in several ways compared to those within the DNA replication restart primosome of *E. coli*. In *E. coli*, the PriA:PriB binary interaction is weak, while the PriB:DNA binary interaction is strong. In *N. gonorrhoeae*, these affinities have been reversed: the PriA:PriB binary interaction is strong, while the PriB:DNA binary interaction is weak.

The crystal structure of *N. gonorrhoeae *PriB provides clues that could account for the low affinity PriB:DNA interaction. Analysis of the binding site for DNA reveals significantly reduced positive electrostatic surface charge potential relative to the analogous surface of *E. coli *PriB, and several aromatic residues of *E. coli *PriB that are known to play a role in binding ssDNA are not conserved in *N. gonorrhoeae *PriB [17,18]. Furthermore, our results indicate that *N. gonorrhoeae *PriB shows little preference for binding specific DNA structures. Thus, it is possible that some portion of the surface of PriB involved in DNA binding in *E. coli *has been adapted for another purpose in *N. gonorrhoeae*, perhaps for interactions with its cognate PriA. This could explain the high affinity PriA:PriB interaction seen in *N. gonorrhoeae *relative to *E. coli*.

Despite variation in the affinities of individual binary interactions within the two bacterial primosomes, we have found that the functional consequences of the physical interactions appear to be similar between the two species in one important way: formation of a PriA:PriB:DNA complex stimulates the helicase activity of PriA. More interesting, however, are the mechanistic details of how this stimulation is accomplished. In *E. coli*, evidence suggests that a ssDNA product-binding mechanism is important for PriB stimulation of PriA helicase activity, likely within the context of a PriA:PriB:DNA ternary complex [7]. Furthermore, PriB has no effect on the rate of PriA-catalyzed ATP hydrolysis in *E. coli *[7]. This indicates that allosteric activation of PriA's ATPase activity is not a key factor in the stimulation of PriA helicase by PriB in *E. coli*.

While we can not rule out a ssDNA product-binding mechanism operating in *N. gonorrhoeae *DNA replication restart, the relatively low affinity with which *N. gonorrhoeae *PriB binds ssDNA suggests that this type of mechanism might not contribute as much to PriB stimulation of PriA helicase activity in *N. gonorrhoeae *as it does in *E. coli*. This hypothesis is further supported by the observation that a *N. gonorrhoeae *PriB variant with greatly diminished ssDNA binding activity can stimulate the helicase activity of PriA at nearly the same levels as does wild type PriB. On the other hand, an allosteric activation mechanism could account for PriB stimulation of PriA helicase in *N. gonorrhoeae*. This form of activation would not necessarily require a high affinity PriB:DNA interaction, and could arise from a conformational change induced in PriA upon binding PriB, thus enhancing the rate at which PriA hydrolyzes ATP and couples ATP hydrolysis to the process of unwinding duplex DNA. An allosteric activation model could also provide a potential functional consequence for the high affinity PriA:PriB interaction observed in *N. gonorrhoeae*.

Despite differences in binary affinities among primosome components, the function of the primosome proteins in these two bacterial species appears to converge on a similar outcome: stimulation of PriA helicase by its cognate PriB. This raises the question of why such differences would have been selected for throughout evolution. One possible explanation lies with the presence of DnaT in *E. coli *and its apparent absence in *N. gonorrhoeae*. In *E. coli*, DnaT is believed to play an important role in primosome assembly and might facilitate the release of ssDNA from PriB within the primosome complex, perhaps making the ssDNA available for binding by the replicative helicase [8,31]. Without the stabilizing weak interactions provided by DnaT, a primosome complex might require an alternate source of weak interactions to achieve an equivalent level of stability. In *N. gonorrhoeae*, a robust PriA:PriB interaction might supply the requisite primosome-stabilizing binding energy that would have otherwise come from DnaT in an organism such as *E. coli*.

Furthermore, the lack of DnaT in *N. gonorrhoeae *could explain the relatively weak affinity with which its PriB binds ssDNA. With no DnaT to facilitate release of ssDNA from PriB, as is thought to occur in *E. coli*, *N. gonorrhoeae *might require its PriB to have an inherently low affinity for ssDNA to promote release of ssDNA without assistance, assuming that PriB actually binds ssDNA in *N. gonorrhoeae *cells. It is possible that some portion of the DNA binding site of *N. gonorrhoeae *PriB has been remodeled to accommodate interactions with its cognate PriA, thereby sacrificing interactions with DNA for enhanced interactions with PriA that could activate PriA's ATPase activity.

Another possible explanation for the differences seen between the two species is that physical interactions among components of the *N. gonorrhoeae *DNA replication restart primosome could have become specialized to meet the physiological demand for DNA replication restart in *N. gonorrhoeae *cells, which likely differs from that in *E. coli *cells. A high affinity interaction between PriA and PriB might indicate that PriA and PriB are constitutively complexed with one another in *N. gonorrhoeae *cells, perhaps facilitating a more rapid response to DNA damage than could be elicited by primosome proteins that must assemble at a site of DNA replication fork reactivation. This type of adaptation could be particularly beneficial for an organism such as *N. gonorrhoeae *that has evolved under selective pressure to withstand relatively high levels of oxidative damage to its genome.

## Conclusions

The results of this study demonstrate that a bacterial PriB homolog with weak single-stranded DNA binding activity can stimulate the DNA unwinding activity of its cognate PriA helicase. While it remains possible that *N. gonorrhoeae *PriB binds DNA in the context of a PriA:PriB:DNA ternary complex, in which the local concentration of DNA could be quite high, our results suggest that *N. gonorrhoeae *PriB might have evolved to interact strongly with PriA instead of with DNA, thus sacrificing high affinity DNA binding for protein:protein interactions with PriA that could modulate PriA's helicase activity. This could account for *N. gonorrhoeae *PriB's ability to stimulate PriA-catalyzed ATP hydrolysis, which is a function not observed with *E. coli *PriA and PriB proteins.

## Methods

### DNAs and proteins

The *priA *and *priB *genes of *N. gonorrhoeae *were cloned and the recombinant PriA and PriB proteins were purified as previously described [17]. DNA substrates used in the equilibrium DNA binding assays and duplex DNA unwinding assays were constructed by annealing complementary DNA oligonucleotides, one of which in each structure was labeled with fluorescein at the 3' end (Table 1). Oligonucleotides were suspended in 10 mM Tris·HCl pH 8, 50 mM NaCl, 1 mM EDTA at a 2:1 molar ratio of non-labeled DNA to fluorescein-labeled DNA. The DNAs were incubated at 95°C for 5 min, slow-cooled to 70°C and incubated at that temperature for 60 min, and slow-cooled to 25°C. Duplex DNAs were gel-purified through 6% polyacrylamide gels using 100 mM Tris borate pH 8.3, 2 mM EDTA as the electrophoresis buffer. The DNAs were excised from the polyacrylamide gels, electroeluted using the same electrophoresis buffer, dialyzed against 10 mM Tris·HCl pH 8, 5 mM MgCl_2_, aliquoted, and stored at -20°C.

### Equilibrium DNA binding assays

Fluorescence polarization spectroscopy was performed at 25°C with a Beacon 2000 fluorescence polarization system (Invitrogen). Serial dilutions of PriA or PriB were made into 20 mM Tris·HCl pH 8, 10% (v/v) glycerol, 50 mM NaCl, 1 mM 2-mercaptoethanol, 0.1 mg/ml bovine serum albumin (BSA) and incubated with 1 nM fluorescein-labeled DNA. Apparent dissociation constants (K_d,app_) were calculated by determining the concentration of either PriA or PriB required to bind 50% of the fluorescein-labeled DNA (Curve Expert 1.3). The unbound state is reported by the fluorescence anisotropy of the fluorescein-labeled DNA in the presence of buffer alone. The fully-bound state is reported by the fluorescence anisotropy of the fluorescein-labeled DNA in the presence of a sufficient concentration of PriA or PriB to saturate the fluorescence anisotropy signal. Data are reported in triplicate and associated uncertainties represent one standard deviation of the mean.

### DNA unwinding assays

DNA substrates were diluted to 1 nM in 20 mM Tris·HCl pH 8, 50 mM NaCl, 3 mM MgCl_2_, 1 mM 2-mercaptoethanol, 1 mM ATP. For unwinding assays involving PriB proteins, indicated concentrations of wild type PriB or PriB:K34A were added to the DNA and incubated for 5 min on ice. Indicated concentrations of PriA were added to the reaction mixtures and incubated at 37°C for 10 min to facilitate duplex DNA unwinding. Reactions were stopped by addition of SDS to a final concentration of 1%. The amount of duplex DNA unwound was determined by measuring the fluorescence anisotropy of the samples following addition of SDS. Fluorescence anisotropy values were compared to the fluorescence anisotropy of the DNA substrate incubated in buffer alone (fully intact DNA substrate) and the fluorescence anisotropy of the DNA substrate after being heated to 95°C and rapidly cooled to 25°C (fully denatured DNA substrate) to calculate the fraction of DNA unwound. Data are reported in triplicate and associated uncertainties represent one standard deviation of the mean.

### ATP hydrolysis assays

PriA-catalyzed ATP hydrolysis was measured using a coupled spectrophotometric assay that has been previously described [32]. This assay uses an ATP regeneration system that converts ADP to ATP in a reaction that is coupled to the conversion of NADH to NAD^+^. The coupled reaction can be monitored spectrophotometrically by measuring the decrease in absorbance at 340 nm due to NADH oxidation. Primosome proteins at indicated concentrations were incubated with indicated concentrations of DNA and ATP in 20 mM Hepes pH 8, 50 mM NaCl, 7 mM 2-mercaptoethanol, 2 mM phosphoenol pyruvate, 0.1 mM NADH, 14 units/ml pyruvate kinase, 20 units/ml lactate dehydrogenase, 0.1 mg/ml BSA at 25°C. Steady-state Δ[NADH]/Δt rates were calculated using the molar extinction coefficient 6,220 M^-1^·cm^-1 ^for NADH, and these rates are equivalent to Δ[ATP]/Δt. The kinetic parameters *K*_m _and *k*_cat _were determined with respect to DNA and with respect to ATP by fitting the ATP hydrolysis rates to the Michaelis-Menten equation,

$$V=\left(V_{\text{max}}\times \;\left[\text{S}\right]\right)∕\left(K_{\text{m}}+\text{ [S]}\right)$$

where S = either DNA or ATP (Curve Expert 1.3). Values of *k*_cat _were determined by dividing *V*_max _by the concentration of PriA in the reaction. Data are reported in triplicate and associated uncertainties represent one standard deviation of the mean.

## Abbreviations

The abbreviations used are: ssDNA: single-stranded DNA; dsDNA: double-stranded DNA; K_d,app_: apparent dissociation constant.

## Authors' contributions

CF, BS, and MEG purified the proteins, constructed the DNA substrates, and carried out the equilibrium DNA binding assays, DNA unwinding assays, and ATP hydrolysis assays. MEL conceived of the study, participated in its design and execution, and drafted the manuscript. All authors read and approved the final manuscript.
